# Can Self-affirmation Encourage HIV-Prevention? Evidence from Female Sex Workers in Senegal

**DOI:** 10.1007/s10461-023-04039-7

**Published:** 2023-05-17

**Authors:** Sara Haire, Aurélia Lépine, Daniel A. Effron, Carole Treibich

**Affiliations:** 1 Institute for Global Health, University College London, London, UK; 2 London Business School, London, UK; 3 Univ. Grenoble Alpes, CNRS, INRAE, Grenoble INP, GAEL, 38000 Grenoble, France; 4 Miami, USA

**Keywords:** Self-affirmation, Self-efficacy, HIV, Stigma, Female sex work, Health

## Abstract

We test an intervention aiming to increase condom usage and HIV testing in a stigmatized population at high risk of contracting HIV: female sex workers (FSWs) in Senegal. Some sex work is legal in Senegal, and condoms and HIV tests are freely available to registered FSWs—but FSWs may be reluctant to get tested and use condoms, in part because doing so would entail acknowledging their risk of contracting HIV and potentially expose them to stigma. Drawing on self-affirmation theory, we hypothesized that reflecting on a source of personal pride would help participants acknowledge their risk of HIV, intend to use condoms more frequently, and take an HIV test. Prior research suggests that similar *self-affirmation* interventions can help people acknowledge their health risks and improve their health behavior, especially when paired with information about effectively managing their health (i.e., *self-efficacy information*). However, such interventions have primarily been tested in the United States and United Kingdom, and their generalizability outside of these contexts is unclear. Our high-powered experiment randomly assigned participants (*N* = 592 FSWs; *N* = 563 in the final analysis) to a self-affirmation condition or a control condition and measured their risk perceptions, whether they took condoms offered to them, and whether (after randomly receiving or not receiving self-efficacy information) they took an HIV test. We found no support for any of our hypotheses. We discuss several explanations for these null results based on the stigma attached to sex work and HIV, cross-cultural generalizability of self-affirmation interventions, and robustness of previous findings.

**Supplementary Information:**

The online version contains supplementary material available at 10.1007/s10461-023-04039-7.

## Introduction

The HIV/AIDS epidemic threatens the health of countless people and undermines efforts to reduce poverty and inequality. In Senegal and other West African countries, where the infection rate ranges from 1 to 4.5% of the population, infections are concentrated among high-risk individuals [1–3]. For example, female sex workers (FSWs) in such countries are up to nine times more likely to be infected with HIV/AIDS than the rest of the population [1–3]. To monitor the prevalence of sexually transmitted infections (STIs) and the spread of the HIV/AIDS epidemic, the Government of Senegal legalized sex work for women older than 21.

FSWs in Senegal have both opportunity and incentive to take certain steps to reduce the spread of HIV. The government requires FSWs to register with a health center, where they must receive subsidized testing and treatment (if applicable) for STIs every month (with a free HIV test offered annually), and where they may take free condoms. A government-issued registration card records these monthly health visits; FSWs who fail to present an up-to-date registration card on demand (e.g., because they are not registered, or have not regularly visited the health center) face a prison sentence of 2–6 months (cf. Penal Code articles 319/325). Thus, FSWs in Senegal not only can protect their health by obtaining free condoms and testing, but they also face legal consequences if they decline to get tested.

However, these opportunities and incentives do not guarantee that people will procure condoms and get tested; psychological barriers may stand in the way. People tend to respond defensively to information that suggests their behavior puts them at risk of negative health outcomes [4]. Downplaying the risk of contracting HIV, FSWs may be reluctant to engage in health-protective behaviors, such as using condoms or getting tested. Moreover, carrying condoms with them or going for a test may draw attention to their membership of a highly stigmatized profession.

Several interventions that encourage condom use and testing in similar populations in other African countries have neglected such psychological barriers. Instead, these interventions provide information about HIV prevention, promote condom use, and distribute free condoms [5–12]. Perhaps, these interventions would be even more effective if they also addressed psychological barriers. The present research tests an intervention targeting two such barriers.

The first psychological barrier is that engaging in HIV-prevention behaviors could require FSWs to admit to themselves that their work puts them at high risk of contracting a deadly disease. Motivated to downplay or ignore this risk, FSWs may be reluctant to procure condoms or get tested. This motivation is not unique to FSWs—aversion to acknowledging their risk status is a common reason people avoid improving their health behaviors and getting screened for disease [13–15]. However, we expect FSWs to be particularly motivated not to acknowledge their risk status, given that it is so much higher for them than for other people.

The second psychological barrier is that sex work in Senegal, though legal, is highly stigmatized. Indeed, prior research identifies this stigma as the main reason Senegalese women who do sex work ignore the legal requirement to register as sex workers [16]. FSWs in Senegal may worry that attending an HIV screening appointment or carrying condoms would, if discovered, signal their stigmatized identity to others. As reminders that their work involves exposure to disease, condoms and testing may also challenge FSWs’ ability to distance themselves from feelings of self-stigma [17]. Indeed, the HIV Prevention 2.0 (HP2) study identified stigma as a barrier to increasing HIV-prevention methods. The study found that decreasing stigma associated with HIV could potentially lead to improvements in health for higher-risk groups such as FSWs [18]. These two psychological barriers are linked by a common principle: health risks and stigma both threaten the fundamental human drive to maintain a sense of self-worth [19].

To address this threat to self-worth, the present research tested a *self-affirmation intervention*, which offers FSWs an opportunity to reflect on a source of personal pride. As we review below, self-affirmation is a well-studied intervention in social- and health-psychology literature. [4, 20, 21] It protects people’s sense of self-worth and can increase their inclination to engage in protective behaviors that acknowledge personal health risks—particularly when paired with information about how to effectively manage their health (i.e., *self-efficacy information*) [20, 22]. Thus, we predicted that self-affirmation could lead FSWs to (a) perceive themselves as being at higher risk of contracting HIV, (b) demonstrate stronger intentions to use condoms, and (c) take an HIV test, particularly after receiving information about how they could effectively manage their health if they were to test positive for HIV.

By testing these hypotheses, we make theoretical and practical contributions to the literatures on health interventions and on self-affirmation theory. On the theoretical side, we examine how psychological barriers may inhibit FSWs’ health-protective behaviors, and we test the generalizability of a self-affirmation intervention to an understudied context. Because most self-affirmation health interventions have been conducted in the United States (US) and United Kingdom (UK), it is unclear how effective such interventions would be in other countries and cultural contexts. Ours is among the first self-affirmation experiments conducted in a low- or middle-income country (cf. [23, 24]). On the practical side, we examine whether a simple, low-cost intervention can reduce risky health behaviors.

Our experiment has several methodological strengths. First, as one of the largest self-affirmation experiments that targets health behavior change, our experiment provides high statistical power to reliably detect even small effects [25]. Second, we test a theoretically and practically important moderator: whether the effect of self-affirmation is stronger when people receive information about how they could effectively manage an HIV-positive diagnosis (i.e., self-efficacy information). Finally, we assess how any effects of self-affirmation cascade through stages of HIV-prevention uptake (i.e., risk perception, intention to change behavior, and behavior change), allowing us to identify potential advantages of incorporating self-affirmation procedures into HIV-prevention policies.

In the next sections, we briefly review the self-affirmation literature and its limitations. After describing our hypotheses and methods, we report the results of a high-powered experiment that finds no evidence that self-affirmation can improve HIV-prevention behaviors among FSWs in Senegal. Finally, we suggest several explanations for these null results, and discuss what they suggest about the limits of self-affirmation interventions.

## Literature Review: Self-affirmation Theory and Health Behavior

According to self-affirmation theory, people are motivated to maintain a global sense of self-integrity [19]—that is, to perceive themselves as a “good and appropriate person” [26]. Yet people also experience threats to this desired self-perception, such as when their behavior exposes them to health risks or stigma. To neutralize these threats, people employ an arsenal of psychological self-defense strategies, such as ignoring or denying health risks [27], or avoiding situations that call attention to their stigma [28]. For example, the fact that smoking causes cancer can threaten smokers’ ability to perceive themselves as “good and appropriate,” which may motivate them to downplay their personal risk of cancer.

Self-affirmation theory’s key insight is that these defensive responses can be reduced by encouraging people to affirm a positive aspect of themselves that is unrelated to the threat [29]. For example, the theory would predict that a smoker would be less motivated to downplay their risk of cancer after thinking about how they are a good parent. Reflecting on their parenting skills (or other source of self-worth) would help the smoker maintain a general view of themselves as a “good and appropriate person,” despite acknowledging that their behavior is harming their health. More generally, self-affirmation bolsters people’s global sense of self-integrity, increasing their ability to weather threats to other aspects of themselves. Leveraging this theoretical insight, self-affirmation interventions have been widely used to improve outcomes in education, intergroup conflict, interpersonal relations, and health [21]. Such interventions typically involve providing an opportunity for people to reflect on a source of pride, an important personal value, or a close relationship [30].

Two findings are particularly relevant for our investigation. The first is that self-affirmation can increase people’s openness to accepting “difficult truths” [31, 32], including that they are at risk of negative health outcomes [25, 33, 34]. For example, randomly assigning smokers to complete a self-affirmation procedure increased how important they thought it was to quit smoking, and increased their likelihood of taking anti-smoking leaflets [35]. Self-affirmation can even increase people’s inclination to engage in protective behaviors that acknowledge their personal health risks. For example, US undergraduates who were randomly assigned to reflect on a personally important (vs. unimportant) value subsequently reported that they were at higher risk of contracting HIV, and took more free condoms at the end of the study [4]. These results comport with the conclusion of a meta-analysis of 144 experimental tests: self-affirmation can increase people’s acceptance of information about health risks, intentions to improve healthy behavior, and actual behavior change [25, 36].

The second key finding is that self-affirmation can help people cope with stigma [37]. For example, Hall et al. [38] recruited participants who experienced stigma from their low socioeconomic status—stigma that could inhibit them from seeking financial help. A self-affirmation procedure increased these participants’ likelihood of taking a leaflet about applying for a tax benefit, suggesting that affirmation helped them cope with stigma. In another study, self-affirmation reduced the stigma that undergraduates associated with psychotherapy, and increased their willingness to seek psychotherapy themselves [28]. In both studies, stigma appeared to inhibit unaffirmed participants from seeking the help they needed, whether for economic or mental health challenges.

In short, prior research suggests that, by bolstering a global sense of self-worth, self-affirmation interventions can diminish people’s reluctance to acknowledge the health risks they face and improve people’s ability to deal with stigma. Earlier, we suggested that such reluctance and stigma are potent psychological barriers in the Senegalese context that they may prevent FSWs from using condoms or getting tested. Thus, we reasoned that self-affirmation has the potential to improve these women’s HIV-prevention behaviors.

There are, however, reasons to doubt whether the salutary effects of self-affirmation found in prior research would emerge among FSWs in Senegal.

First, most research has been conducted in the US and UK (see Appendix S1 for examples), two of the most individualistic countries in the world [39]. Self-affirmation procedures, such as reflecting on a source of personal pride, may represent a less culturally sanctioned activity in more collectivistic cultures such as Senegal.

Second, Senegalese FSWs face greater health risks and are more stigmatized than most of the populations previously studied in the literature. Most research in this literature was conducted with undergraduates whose unhealthy behaviors were associated with little or mild stigma, such as consuming alcohol [33, 40, 41] or caffeine [33], smoking [35], or failing to use sunscreen [20]. It is possible that a standard self-affirmation procedure would be insufficient to overcome much-greater stigma that FSWs in Senegal face. Moreover, even in the populations previously studied, self-affirmation has at times increased defensive responses, particularly among participants with greater health risks [33, 41].

Despite these doubts, we hypothesized that self-affirmation would improve HIV-prevention behaviors among FSWs in Senegal. We next describe our specific hypotheses and how we tested them.

## Hypotheses and Methodology

### Research Hypotheses

Our experiment tested whether self-affirmation could encourage FSWs in Senegal to participate in HIV-preventive measures. Our key outcome measures include taking free condoms and getting an HIV test because condoms and testing are the main preventive tools available in the fight against HIV. Given that the consistent use of condoms is the most cost-effective way to prevent HIV transmission [42, 43], condom use is the central pillar of any HIV-prevention strategy in most countries. HIV screening confers direct benefits to both the tested person and her sexual partners. Those who test positive will be referred for treatment, which will reduce viral load and curb HIV transmission [44]. However, absent self-affirmation, participants in our experiment might be reluctant to take condoms and get tested, in part because these behaviors (a) involve acknowledging their risk of contracting an STI, and (b) highlight their membership of a stigmatized profession (e.g., carrying condoms with them after the study could identify them as sex workers).

In overview, our experiment proceeded as follows. We randomly assigned half the participants to discuss an experience that made them feel proud. We then measured all participants’ risk perception, intention to use condoms, and whether they accepted an opportunity to take free condoms. Next, orthogonally to the self-affirmation manipulation, we randomly assigned half the participants to receive information about how HIV can be medically managed following a positive HIV test (i.e., providing them with *self-efficacy information*). This manipulation allowed us to test whether the effects of self-affirmation on subsequent outcome measures would be stronger when people believe they can effectively manage their health [22]. The final outcome measures were whether participants signed up for an HIV-screening appointment, and (if so) whether they showed up for this appointment.

We hypothesized that, when compared to the control group, FSWs in the self-affirmation condition would:

- *Hypothesis 1* (H1): perceive that they faced greater health risks (i.e., rate themselves as being more likely to have HIV or an STI at present or in the future; be more likely to acknowledge that they did not previously practice safe sex).
- *Hypothesis 2* (H2): be more likely to take steps to use condoms in the future (i.e., express stronger intentions to use condoms, be more likely to take condoms, and take a larger number of condoms).
- *Hypothesis 3* (H3): be more likely to sign up for and then later take an HIV test.

In short, we predicted that self-affirmation would improve acceptance of information about health risks, intentions to improve healthy behavior, and actual behavior [25].

We also expected that providing the FSWs with information about the benefits of HIV testing would magnify the effect of self-affirmation on the likelihood of signing up and showing up for an HIV test:

- *Hypothesis 4* (H4): the effect predicted by H3 will be particularly large among FSWs provided with self-efficacy information.

### Recruitment of Participants

In August 2017, we invited the 651 FSWs from a cohort created in 2015 to attend a survey interview at a health facility. Inclusion criteria required participants to be active FSWs and to be over 21 years old. Note that this sample was stratified by the registration status of the FSWs and as a result included an equal proportion of registered and unregistered sex workers. Registered FSWs were recruited by midwives based on their hospital file from four (out of five) STI centers located in suburbs of the capital Dakar (Rufisque, Pikine, Mbao, and Sebikotane).

Non-registered FSWs were recruited by non-registered peer leaders. Out of the 651 FSWs, 441 agreed to participate, and the cohort was replenished with an additional 150 FSWs using the same method of recruitment as for the initial cohort. As a result, 591 participated in the study. However, 28 were excluded from the analysis due to inconsistencies in their responses and understanding of the probability questions; 563 participants were included in the final analysis.

Interviews were conducted in separate rooms by professional interviewers. Ethical clearance was obtained from a higher education institution in the UK and from the national ethics committee in Senegal. Written consent was collected from participants in order for them to participate in the study. Data were stored on a secured server, and anonymized data were used for data analysis.

### Experiment

#### Design and Overview

The experiment had a 2 × 2 sequenced factorial design (self-affirmation vs. no self-affirmation) × (self-efficacy information vs. no information). In overview, we administered the self-affirmation manipulation, asked the participants to respond to dependent measures assessing risk perceptions and condom use, administered the self-efficacy information, and then assessed our final dependent measures: whether participants signed up for and showed up to take an HIV test. Note that because the self-efficacy manipulation was specifically designed to motivate HIV testing (i.e., it provided information about the benefits of such tests), we placed this manipulation just before the measures of HIV testing but after the other dependent variables. As a result, we can only test the effect of the self-efficacy manipulation on the uptake of HIV testing.

#### Procedure and Experimental Manipulations

Participants were invited to the health facility and were interviewed in private rooms. The survey lasted 1.5 h and was conducted by interviewers who recorded responses on electronic devices. The interviewers were trained to follow a standardized script to ensure that the experiment followed the same procedure at different health centers (see Appendix S2.1 for full script).

At the beginning of the study, participants answered questions about whether they were registered as sex workers, their income from various sources, and how opposed to risk they were. They provided information about their relationships with their clients, their marital status, their condom use, and several related questions. These measures were not related to our hypotheses about self-affirmation and self-efficacy, so we do not discuss them further.

Next, we administered the self-affirmation manipulation adapted from Hall et al. [38]. Participants who were randomly assigned to the self-affirmation condition were asked to discuss an experience that made them proud, or when they achieved a goal that was close to their heart. To encourage all participants to speak for several minutes, the interviewers asked open-ended questions if the participant fell silent (“It’s interesting, can you tell me more?”; “How did you feel at that moment?”; “What does this tell us about you?”). Participants randomly assigned to the no self-affirmation condition were not asked to discuss anything.

Participants then responded to a series of outcome measures (described in the next section; see also Table 1). Specifically, they answered a series of questions to assess their risk perceptions and behavioral intentions to practice safe sex, and were given an opportunity to take some free condoms from a bowl.

**Table 1:** Outcome measures used

Risk perceptions
What is the probability of having HIV OR an STI today? (0–100%)^^^
What is the probability of being HIV+ 1 year from now? (0–100%)
What is the probability of contracting HIV from someone who is HIV+ OR has STI by having unprotected sex? (0–100%)^^^
What is the probability of using a condom for next paid sex act? (0–100%)
What is the probability of using a condom for next 10 paid sex acts? (0–100%)
How many of the last 5 sex acts were protected? (0–100%)
Plans to use condoms
Any condoms left in bowl (1 if yes, 0 otherwise)
Took any condoms (1 if yes, 0 otherwise)
Number of condoms left in bowl (max 20, min 0)
HIV testing
Wanted to take an HIV test that day (1 if yes, 0 otherwise)
Whether the participant took an HIV test that day at the clinic (1 if yes, 0 otherwise)

*Note: *All measures in the section used 0–100% and those who did not understand probability were not included in this part of the analysis (participants were asked the probability of it raining during the rainy season and those who said 20% or less were excluded from this portion, as well as those who explicitly said they did not understand probabilities); 43 women were excluded in total based on their understanding; a further 22 were excluded when they reported nothing to be proud of

^^^Indicates two variables that were highly correlated (> 0.72), so were averaged and combined

Then, depending on random assignment to a self-efficacy condition, participants either did or did not receive information about the benefits of HIV screening. The survey interviewer summarized these benefits by explaining, “If a person gets screened and is infected, she can take antiretroviral therapy that prevents the disease from developing, which improves her quality of life and prevents the transmission of the virus to other people.” (See Appendix S2.1 for full script.) We reasoned that receiving such information should increase participants’ belief that they would be capable of managing an HIV infection (i.e., increasing their self-efficacy with respect to managing HIV).

#### Outcome Measures

We expected that self-affirmation would encourage participants to acknowledge their high risk of contracting HIV, form intentions to engage in preventive behaviors (e.g., plan to use condoms), and then engage in preventive behaviors (e.g., sign up for and attend an HIV screening).

#### Risk Perceptions

We included several measures of risk perceptions to test H1 (i.e., that self-affirmation would increase risk perceptions). First, participants were asked to rate out of 100 how likely they thought it was that they were HIV-positive or had another STI at the time of the study. Participants were given training on the scale and checks for understanding were performed before measuring the subjective expectations of the participants. Next, three items assessed participants’ perceptions of contracting HIV in the future: the probability that: (a) they would be HIV-positive in a year; (b) they would contract HIV from unprotected sex with an HIV-positive person; and (c) they would contract HIV through unprotected sex with an HIV-positive person, given they already had an STI. Finally, we asked how many times participants had used condoms in their last five sexual encounters, based on the logic that reporting more frequent condom use would suggest greater acknowledgement of HIV/STI risk. The reported probability of contracting HIV from someone who is HIV-positive and the probability of contracting an STI through unprotected sex were highly correlated, and were thus combined and averaged (Table 1).

#### Planned Condom Use

To test H2—that self-affirmation would lead people to take more steps to use condoms in the future—we administered self-report measures and a behavioral measure of intended condom use. The self-report items asked participants how likely they were to use condoms for their next 10 paid sex acts. For the behavioral measure, the interviewer gestured to a bowl of condoms, inviting participants to take as many free condoms as they would use. If participants took none, they were asked why. At the end of the study, after participants had left the room, the interviewer counted the number of condoms left in the bowl, recording the number the participants had taken.

#### HIV Testing

To test our final two hypotheses—that self-affirmation would increase people’s likelihood of signing up and showing up for an HIV test (H3), particularly after receiving self-efficacy information (H4)—we first asked participants whether they wanted to take an HIV test that day (yes or no). Then, to measure behavior change, we followed up with the health clinic to see who did in fact take an HIV test that day.

#### Other Measures

As an exploratory analysis, we wanted to examine whether the effect of self-affirmation would depend on the type of experience that participants said made them feel proud. Based on participants’ answers, we constructed a list of categorical variables associated with the specific proud moments the women mentioned. These included: paying for or arranging a baptism or wedding; taking care of family; having children; receiving a gift; paying for furniture, vacations, or objects for themselves; and other significant proud moments. Some participants did not mention feeling proud of anything, which comprised a seventh category. We also recorded how long each participant discussed the experience that made them feel proud, to explore whether any self-affirmation effects would be stronger among people who spent more time engaged in the affirmation exercise.

#### Empirical Strategies

For the main analysis, we estimated joint tests of significance and *t*-tests, and logit and tobit regression models, depending on the characteristics of the outcome considered, to determine whether receiving the self-affirmation manipulation made FSWs more likely and willing to accept threatening health information, intend to change behavior, and subsequently change behavior. For the measure of whether participants took any condoms from the bowl (binary variable), we estimated:

$$Logit\left( {Y_{i} } \right) = \alpha _{0} + \alpha _{1} *Self{\text{-}}affirmation$$

For the measure of whether a participant agreed to be tested for HIV, and whether she actually took the test (binary variables):

$$Logit(Y_{i} ) = \alpha _{0} + \alpha _{1} *Self{\text{-}}affirmation + \alpha _{2} *Self{\text{-e}}fficacy\,Information + \alpha _{3} (Self{\text{-}}affirmation*Self{\text{-}}efficacy\,Information)$$

where *Y*_*i*_ corresponds to the outcomes of interest that can take the value of *0* or *1*. Self-affirmation and self-efficacy information is coded *1* if the participant was self-affirmed or received self-efficacy information, respectively, and *0* otherwise; their respective effect is estimated by taking the average marginal effect of the index coefficients *α*_1_ and *α*_2_. In addition, we test the effect of receiving both the self-affirmation and self-efficacy information by treatments by interacting these variables, estimating the average marginal effect from *α*_3_.

To model the number of condoms left in the bowl, we used a tobit specification with a lower censor of 0 and an upper censor of 20, the maximum number of condoms contained in the bowl. We also used a tobit model to analyze responses to the questions about risk perception since these data are censored between 0 and 100.

For the risk-perception questions and the number of condoms taken from the bowl:

$$Tobit(Z_{i} ) = \beta \alpha _{0} + \beta _{1} *Self{\text{-}}affirmation$$

where *Z*_*i*_ corresponds to either the probability (ranging from 0 to 100) or the number of condoms taken from the bowl (ranging from 0 to 20).

We also conducted an exploratory sub-group analysis, using a logit regression model and a tobit regression model, to estimate whether any effects of the self-affirmation manipulation on the condom use and HIV test measures depended on the specific proud moment that participants discussed, or on how long they spent discussing their proud moments.

We analyzed the data using STATA Intercooled version 15.1 statistical software (StataCorp, 2015). The experiment and the data analysis plan were pre-registered at https://aspredicted.org/BG9_J16. We did not deviate from the pre-registered plan.

## Results

### Participant Characteristics and Randomization

**Fig. 1:**
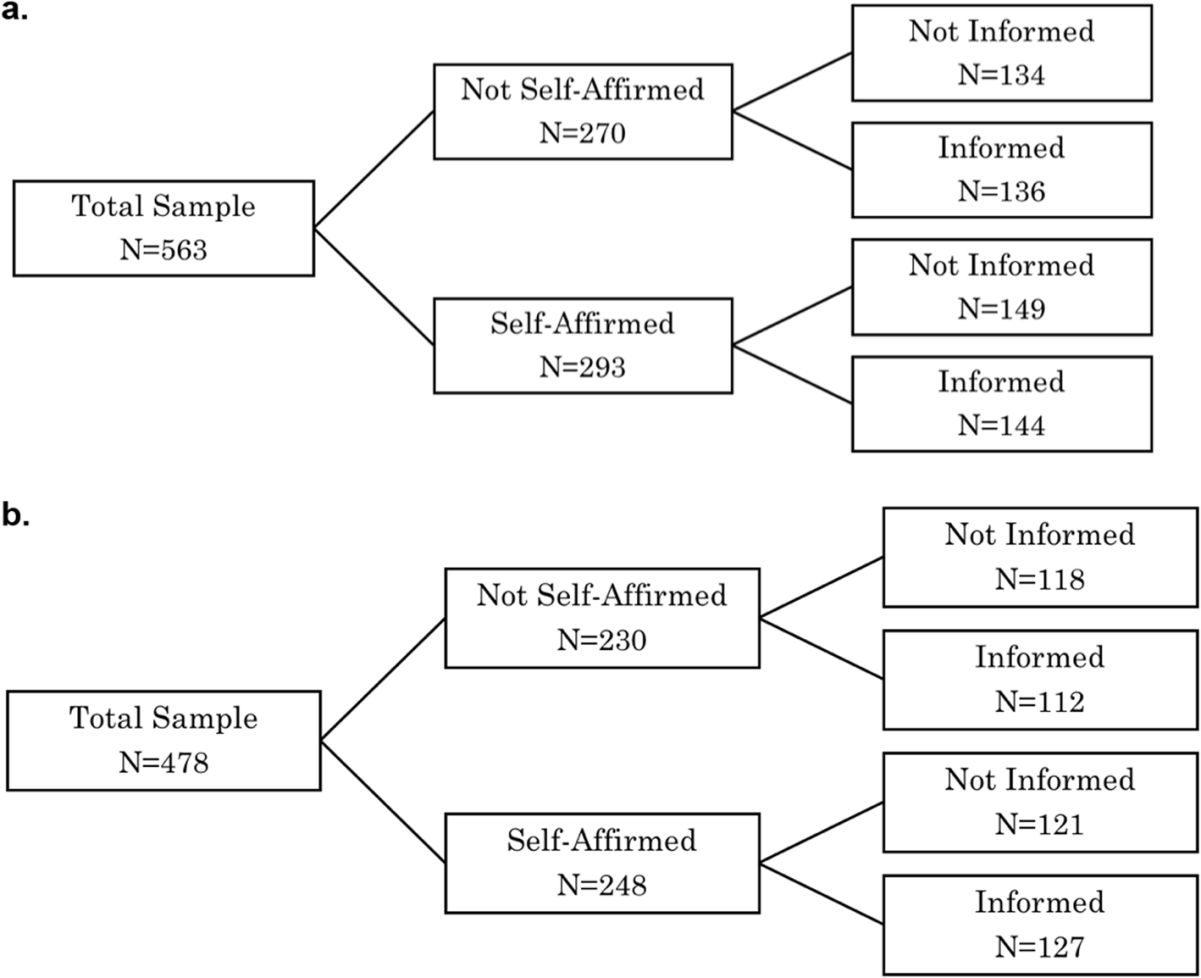
Number of observations per treatment groups. **a** Total sample. **b** Excluding inactive and HIV-positive FSWs

Figure 1 presents the number of observations per group. We estimate this for the whole sample (excluding participants who incorrectly answered our comprehension-check questions on probability) but also on the sample of HIV-negative and active FSWs. We focus on these women because the intervention was targeted at active sex workers and trying to get women to take part in HIV-prevention methods; the intervention may not affect those who are HIV-positive.

For the four different groups, we performed a series of *t*-tests and two tests of joint significance on variables associated with important characteristics of FSWs. We found that the randomizations were successful.

The descriptive statistics of the sample are reported in Table 2; means are presented with SE in brackets for continuous variables. We see that 87.9% of the FSWs were active at the time of the survey. They were, on average, 38.7 years old and averaged 3.5 occasional clients a week. Some 87.0% said they would be ashamed if a neighbor saw them soliciting; 62.5% feared a discriminatory attitude toward being HIV positive and 66.2% feared discriminatory attitudes toward their sex work; and only 30.6% had at least one family member who knew that they were involved in sex work. Some 83.0% of the FSWs had been tested for HIV in the last year and reported an average monthly revenue of 128,022 CFA francs (~ 218 USD at the time of the survey) from sex work. On average, 66.4% were divorced or separated, and 18.5% had never been married. For descriptive statistics on the population that exclude HIV-positive and inactive sex workers, see Appendix S3.

**Table 2:** Descriptive statistics

Mean [Std. Error for continuous variables]	Total sample	Not self-affirmed		Self-affirmed	
		Not informed	Informed	Not informed	Informed
Socio-demographic characteristics	*N* = *563*	*N* = *134*	*N* = *136*	*N* = *149*	*N* = *144*
Age (in years)*	38.73 [9.53]	38.6 [0.75]	38.44 [0.82]	38.72 [0.80]	39.14 [0.83]
Is divorced or separated (%)*	66.4	67.9	66.2	62.4	69.4
Never married (%)*	18.5	17.2	19.9	20.8	16.0
Married (%)°	5.7	5.2	4.4	6.7	6.3
Widowed (%)°	9.4	9.7	9.6	10.1	8.3
Uses contraceptive method (%)*	64.7	67.2	63.2	61.7	66.7
Uses condoms as contraceptive method (%)*	22.7	23.1	26.5	20.8	20.8
HH monthly expenses*	357,237 [12,395]	350,568 [23,409]	375,668 [29,317]	352,704 [21,460]	350,727 [24,981]
Monthly sex revenues	128,022 [5041]	129,237 [9704]	122,094 [12,078]	124,873 [8912]	135,421 [9676]
Household received transfer from migrants in last year (%)°	24.8	20.9	23.9	28.9	25.2
Household sent transfer to migrants in last year (%)°	26.3	26.9	20.7	27.5	29.9
Life satisfaction (scale 1–5 (very dissatisfied to very satisfied))	3.18 [0.04]	3.11 [0.08]	3.18 [0.08]	3.15 [0.09]	3.27 [0.07]
Health status (100–0% (best to worst))*	77.68	77.84	79.26	77.34	76.39
Feeling of helplessness with daily issues*	58.6	62.7	55.1	58.4	58.3
Fear of discriminatory attitudes toward HIV (%)	62.5	66.1	59.3	58.2	66.7
Fear of discriminatory attitudes toward sex work (%)	66.2	66.7	68.4	63.3	66.7
At least 1 family member knows about her sex work (%)°	30.6	36.1	30.7	33.9	22.2
Ashamed if neighbour sees her soliciting (%)°	87.0	81.5	83.2	92.1	90.6
Sex work activity					
Active sex worker (%)	87.9	89.6	87.5	85.2	89.6
Last client was a regular client (%)*	72.7	71.7	78.2	70.1	71.3
Declared use of condom with last client (%)*	96.8	95.8	98.3	98.4	94.5
Number of occasional clients in a week*	3.52 [0.26]	3.81 [0.51]	3.59 [0.60]	3.80 [0.55]	2.92 [0.43]
Price of last sex act*	17,002 [1645]	22,367 [5998]	14,887 [1320]	14,165 [1011]	16,754 [2478]
Link with the authorities and the health systems					
Registered sex worker (%)°	50.4	52.5	51.3	48.0	50.0
Police violence in last year (%)*	5.2	5.3	3.7	5.4	6.3
Is affiliated with STI centre (%)°	57.2	60.6	52.2	57.7	58.3
Came to an STI center in the last month (%)*	32.9	32.1	27.9	34.9	36.1
Had HIV screening in past year (%)*	82.9	79.1	85.3	83.2	84.0
Participated in the PrEP project (%)*	19.4	17.9	16.9	24.2	18.1
Outcomes					
Took an HIV test at the center (%)*	37.2	39.6	37.8	33.3	38.3
Wanted to take an HIV test that day (%)*	44.9	45.5	47.8	38.3	48.6
Any condoms left in bowl (%)*	37.5	31.6	41.2	39.6	37.5
How many condoms left in bowl*	6.43	5.67	6.81	6.74	6.46
Number of minutes women spent discussing proud event	2.86	N/A	N/A	2.94	2.77
Test of joint significance					
Self-affirmation					
Considering the variables indicated by *	F(21, 373) = 0.59, *p*-value = 0.9252				
Considering the variables indicated by * and °	F(28, 373) = 0.93, *p*-value = 0.5714				
Self-efficacy information					
Considering the variables indicated by *	(22, 170) = 1.11, *p*-value = 0.3365				

*, °were denoted for the test of joint significance

### Descriptive Statistics of the Self-affirmation Manipulation

**Fig. 2:**
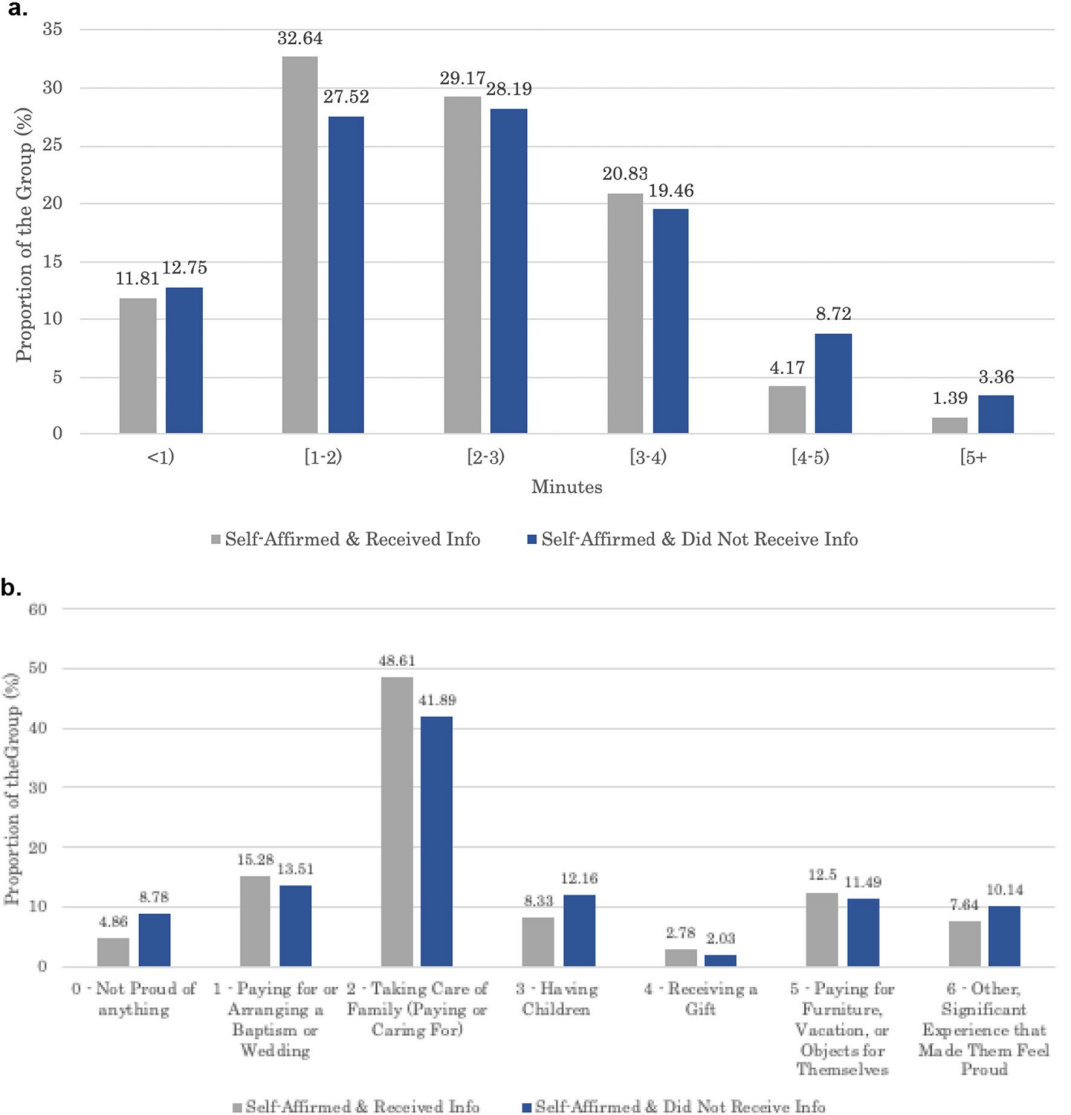
Characteristics of the self-affirmation experiment. **a** Time spent speaking (by group). **b** Experiences that made participants feel proud (by group)

On average, participants in the self-affirmation condition spoke for 2.86 min about an event that made them feel proud. Figure 2a displays a histogram of the number of minutes they spoke for. It shows that most participants spoke for between 2 and 4 min.

In addition, Fig. 2b shows that what participants spoke about fell into seven topic areas: not proud of anything; paying for or arranging a baptism or wedding; taking care of family; having children; receiving a gift; paying for furniture, a vacation, or objects for themselves; and other significant proud moments. Paying for or caring for family was the most common type of experience participants discussed.

### Effect of Self-affirmation on HIV-Prevention Uptake

None of our hypotheses received support (see Table 3). Specifically, we found no evidence that self-affirmation significantly affected: (a) any of the four measures of risk perception (H1); (b) intention to use condoms or likelihood of taking condoms during the survey session (H2); or (c) signing up and showing up for an HIV test (H3). Moreover, the likelihood of signing up and showing up for an HIV test did not significantly depend on the self-efficacy manipulation (H4).

**Table 3:** Average marginal effects for outcome measures

	Risk perception (hypothesis 1)						Plans to use condoms (hypothesis 2)			HIV testing (hypothesis 2 and 3)	
	(1)	(2)	(3)	(4)	(5)	(6)	(7)	(8)	(9)	(10)	(11)
	Probability of having HIV OR an STI today^^^	Probability of being HIV+ in 1 year	Probability of contracting HIV from someone who is HIV+ OR has STI by having unprotected sex^^^	How many of the last 5 sex acts were protected	Probability of using a condom for next paid sex act	Probability of using a condom for next 10 paid sex acts	Any condoms left in bowl	Took any condoms	Number of condoms left in bowl	Wanted to take an HIV test	Took an HIV test that day
Total population											
Self-affirmed	0.0143	1.383	1.318	0.0558	− 1.962	− 1.331	0.0214	0.0190	2.599	− 0.0735	− 0.0627
	(0.01)	(0.60)	(0.76)	(0.99)	(− 1.02)	(− 1.01)	(0.52)	(0.46)	(0.49)	(− 1.24)	(− 1.09)
Received info										0.0224	− 0.0174
										(0.37)	(− 0.30)
Self-affirmed*										0.0815	0.0678
Received info										(0.98)	(0.83)
*N*	522	522	520	495	454	453	562	563	563	563	557
Excluding inactive and HIV positive FSWs											
Self-affirmed	0.441	1.491	0.468	0.0873	− 1.250	− 0.769	− 0.00665	− 0.00853	− 0.614	− 0.0361	− 0.00665
	(0.24)	(0.64)	(0.25)	(1.33)	(− 0.64)	(− 0.56)	(− 0.16)	(− 0.20)	(− 0.12)	(− 0.56)	(− 0.16)
Received info										0.0677	− 0.0463
										(1.04)	(− 0.73)
Self-affirmed*										0.0390	0.0231
Received info										(0.43)	(0.37)
*N*	464	464	463	454	454	453	506	497	497	478	473

***p < 0.01, **p < 0.05, *p < 0.1

^^^Indicates two variables were averaged if they were highly correlated. Columns 1–6, and 9 are the average marginal effects from a tobit regression; and columns 7–8 and 10–11 are the average marginal effects from a logit regression. *t* statistic in parentheses

### Exploratory Sub-group Analyses

Next, we explored whether the effect of self-affirmation on condom use or HIV testing depended on the specific kind of proud moment that participants discussed. The results showed few systematic differences (see Appendix S4). The only significant effect was that participants who discussed taking care of family for the self-affirmation exercise were less likely to leave any condoms in the bowl among the total population and two different comparison groups (those who discussed an experience that made them feel proud compared to the rest of the participants, and those who discussed a particular experience that made them feel proud compared to those who discussed being proud of something else).

Finally, we found no evidence that people in the self-affirmation condition who spent longer talking about a proud moment (and thus may have been “more self-affirmed”) responded differently on the measures of condom use or HIV testing than participants who spoke for shorter amounts of time, or participants who were not self-affirmed. These results were not significantly moderated by the self-efficacy-information manipulation (see Appendix S5). In addition, we find that the results are not driven by the fact that some FSWs might have already taken an HIV test recently (Appendix S6), or because registered FSWs might have better access to condoms and tests (Appendix S7).

## Discussion

Despite numerous studies documenting the salutary effects of self-affirmation on health outcomes, our large-sample study found no evidence that self-affirmation led FSWs in Senegal to accept threatening information about their risk of contracting HIV (H1), to take steps toward practicing safer sex (e.g., by accepting an offer of free condoms) (H2), or to sign up and show up for an HIV test (H3)—even when the affirmation was accompanied by self-efficacy information about the benefits of HIV testing (H4). The null effect of self-efficacy information could reflect the possibility that FSWs already know this information.

We consider several potential explanations for the null effects of self-affirmation. Like any null effects, the present results cannot prove that self-affirmation had no effect, because it is possible that any effect size was too small for our study to detect. However, our study’s large sample size allows us to conclude that if self-affirmation does affect our outcome measures in this population, the size of such an effect is likely to be small. Ours was one of the largest studies of self-affirmation and health behaviors; for example, fewer than 8% of the studies in Sweeney and Moyer’s meta-analysis [36] had sample sizes larger than 100, whereas our study had 563 participants included in this analysis (591 in the entire study). Thus, we do not view low statistical power as a likely explanation for our null results.

Another explanation is that the self-affirmation manipulation failed to make participants feel affirmed. Although this specific manipulation—asking participants to reflect on a source of personal pride—is common in the self-affirmation literature [31, 38, 45, 46], the manipulation has not, to our knowledge, been tested in an African cultural context. Note, however, that over 96% of participants in the self-affirmation condition could think of a time when they felt proud, and our conclusions remained unchanged when we reran the analyses with the 4% of participants who could not.

Perhaps cultural factors explain our results. As noted, most studies on self-affirmation and health have been conducted in the US and UK, which are two of the most individualistic countries in the world, whereas the present study was conducted in Senegal, which has a more collectivistic culture [39, 47]. Of relevance to self-affirmation, individualistic cultures encourage people to conceptualize the self as defined by internal attributes, distinct from social context. People in such cultures tend to adopt an *independent model of self* [48, 49]. By contrast, collectivistic cultures encourage people to conceptualize the self as defined by roles and relationships, and inextricably embedded in social contexts—an *interdependent model of self* [48, 49]. On one hand, asking participants to speak about a source of personal pride may have been tantamount to encouraging them to affirm an *independent* self. In that case, perhaps a more effective treatment would have been to encourage affirmation of an *interdependent* self [29, 50]; for example, by asking participants to think about a time they felt proud to be part of a group or community [51, 52]. On the other hand, a large plurality of participants in our self-affirmation condition spoke about a time when they helped friends and family, a topic that in theory should affirm an interdependent self [53]. Thus, it is not obvious that our self-affirmation task was culturally inappropriate in Senegal.

Another explanation we considered concerns the high degree of stigma associated with sex work and HIV in Senegal [13, 16]. In theory, self-affirmation should help people cope with stigma by bolstering their sense of self-worth [37]. However, the intensity of the stigma that our participants face in their daily lives may simply be too great for a brief self-affirmation task to attenuate, even momentarily. Moreover, the stigma these participants face can have serious consequences. More than half of the FSWs surveyed were fearful of discrimination toward sex work and being HIV-positive (66.2% and 62.5%, respectively, as seen in Table 2). Even if our self-affirmation manipulation reduced participants’ feelings of self-stigma, they would still have cause to worry about backlash from their families or community if they were found carrying condoms or observed getting an HIV test. Without addressing these potential social consequences, self-affirmation may be ineffective.

It is possible that the self-affirmation intervention did not significantly increase the uptake of condoms because participants already had enough condoms. Indeed, when we asked participants who left some condoms in the bowl why they did so (*N* = 222), the majority replied that they had condoms at home. However, this explanation cannot account for why the intervention did not significantly affect intentions to *use* condoms. Similarly, it is possible that self-affirmation did not increase uptake of HIV testing because participants had recently been tested. Indeed, 82.9% of our participants had taken an HIV test within the last 12 months. However, we believe that previous testing would not have discouraged FSWs from getting tested in our experiment given that they are frequently exposed to the risk of contracting HIV. It is also important to note that there are no legal consequences for registered FSWs who test positive for HIV, so the registration status should not act as a barrier to taking an HIV test.

Finally, our intervention may not have improved HIV-prevention behaviors because factors other than psychological barriers have a large influence on these behaviors. For example, FSWs do not simply decide themselves whether to use condoms; they must bargain with clients, who are generally willing to pay more for unprotected sex [54]. Our intervention was not designed to help FSWs overcome pressure from clients and financial incentives against condom use.

In short, although more research would be needed to understand exactly why self-affirmation did not significantly improve health behaviors among FSWs in Senegal, or whether the same results would emerge in other populations, our findings raise the possibility that self-affirmation may not be the best tool to improve health in highly stigmatized populations.

Our results highlight limitations of prior research on self-affirmation. On one hand, positive effects of self-affirmation have been documented in diverse domains and contexts [21]. Self-affirmation interventions can help lift the educational achievement of racial minority groups [26, 21], alleviate intergroup conflict [31, 55], and—of greatest relevance to the present research—improve health behaviors [25]. On the other hand, our research sounds a note of caution. A brief affirmation intervention will not always be an effective way of promoting healthy intentions and behaviors, even in contexts like the present one where there are a priori reasons to believe that it should. As noted, prior work suggests that affirmation improves health behaviors by helping people to cope with stigma and acknowledge their vulnerability to negative health outcomes. But although FSWs in Senegal face substantial stigma and risk of HIV infection, we found no evidence that self-affirmation increased their acknowledgement of this risk or improved their intentions and behaviors. More research is needed to understand why, because there are many differences between the Senegalese FSWs in our study, and US and UK participants in previous studies. Nonetheless, our findings suggest that the effects of self-affirmation on health may not be as broadly generalizable as current theorizing would suggest.

These findings thus highlight the need for more research on the effects of self-affirmation in diverse populations. Like most theories in social psychology, self-affirmation theory was developed mainly using research on participants from Western, educated, industrialized, rich, and democratic (i.e., “WEIRD”) societies that represent only a minority of the world’s population [29, 56]. Without additional data, we should not assume that self-affirmation works in the same way outside of these contexts [57]. It is our hope that the present research sparks more interest in testing whether, when, and why self-affirmation can improve health outcomes among highly stigmatized, at-risk individuals in low- and middle-income countries.

## Conclusion

Self-affirmation holds promise as an intervention for improving health outcomes [25], but prior research leaves questions about how well such an intervention would work beyond the US and UK. Our large-sample field experiment in Senegal found no evidence that reflecting on a source of pride increased female sex workers’ acknowledgement of their risk of contracting HIV, their intention to use condoms, or their likelihood of taking an HIV test. These results raise questions about the generalizability of previous findings, highlighting the need for more research on self-affirmation and health behaviors in highly stigmatized, at-risk populations in low- and middle-income countries.

### Supplementary Information

Below is the link to the electronic supplementary material.

Supplementary file1 (DOCX 3180 KB)
