# Supplementary material for: Can Self-affirmation Encourage HIV-Prevention? Evidence from Female Sex Workers in Senegal
Source: AIDS Behav. 2023 May 17;27(10):3183–96. doi: 10.1007/s10461-023-04039-7 (PMC10516782; doi:10.1007/s10461-023-04039-7)
Supplement: Supplementary file 1 — Supplementary file1 (DOCX 3180 KB) [file 10461_2023_4039_MOESM1_ESM.docx]

**Appendix S1: Sample of Self-Affirmation Studies on Health Behaviour Change**

| First Author, year | Health behaviour | Intention (N) | Behaviour (N) | Type of affirmation | Location | Type of participants |
| --- | --- | --- | --- | --- | --- | --- |
|  |  |  |  |  |  |  |
| Reed (1998) | Caffeine intake | Yes (33) | Yes (33) | Kindness | US | Student |
| Sherman (2000)  Study 2 | Condom use | No | Yes (61) | Value | US | Student |
| Harris (2005) | Alcohol | Yes (36) | Yes (31) | Value | UK | Student |
| Dillard (2005) | Smoking | Yes (130) | No | Value | US | Student |
| Armitage (2008) | Smoking | Yes (57) | Yes (57) | Kindness | UK | Nonstudent |
| Epton (2008) | Fruit & veg intake | Yes (93) | Yes (87) | Kindness | UK | Student |
| Jessop (2009) | Sunscreen | Yes (82) | Yes (82) | Kindness | UK | Nonstudent |
| Meier (2010) | Alcohol | Yes (90) | Yes (90) | Value | US | Student |
| Armitage (2011) | Alcohol | No | Yes (185) | Kindness | UK | Nonstudent |
| Good (2011) | Sunscreen | Yes (170) | No | Value | UK | Student |
| Klein (2011)  Study 2 | Caffeine intake | Yes (50) | No | Value | US | Student |
| Lannin (2013) | Psychotherapy | Yes (84) | Yes (84) | Value | US | Student |
| Jessop (2014)  Study 1 | Exercise | Yes (67) | Yes (54) | Kindness | UK | Students/  Nonstudents |
|  |  |  |  |  |  |  |

Note. See Epton et al *The Impact of Self-Affirmation on Health-Behavior Change: A Meta-Analysis* supplemental material.

**Appendix S2: Script and setting of the experiment**

**Appendix S2.1. Script**

***For the affirmed group***

- Could you tell me about an experience in which you felt proud, when you achieved a goal that was close to your heart?

ENUMERATOR: The FSW must speak for several minutes. You can **restart** the respondent by saying: “it’s interesting, can you tell me more?”; “How did you feel at that moment?”; “What does this tell us about you?”.

ENUMERATOR: Look at the time the respondent starts talking and when she stops. This in order to be able to estimate the time of the discussion.

READ: Thank you for telling me about this experience.

- ENUMERATOR: Did the person think long (more than 15 seconds) before finding an event? She responded directly/ She reflected a few moments (less than 15 seconds) and then responded/ She had to think more than 15 seconds before finding what to talk/ She did not know what to say

- ENUMERATOR: What did the respondent talk about?

- ENUMERATOR: How long the person talk about this experience? Less than 1 minute/ Between 1 and 2 minutes/ Between 2 and 3 minutes/ Between 3 and 4 minutes/ Between 4 and 5 minutes/ More than 5 minutes

***For the non-affirmed group***

No question is asked to the neutral group, you go directly to the next section.

***Expected probabilities of infections***

For the questions below, please use the following scale:

- What is the probability that you have a STI other than HIV/AIDS today? Value between 0 and 100

- What is the probability that you are HIV positive today? Value between 0 and 100

- What is the probability you will be HIV positive in one year from now? Value between 0 and 100

- If you have an UNPROTECTED SEX ACT with a HIV positive person, what is the probability that you will be infected too after this sex act? Value between 0 and 100

- If you have an UNPROTECTED SEX ACT with a HIV positive person and that you have a STI, what is the probability that you will be infected too after this sex act? Value between 0 and 100

- If you have an PROTECTED SEX ACT with a HIV positive person, what is the probability that you will be infected too after this sex act? Value between 0 and 100



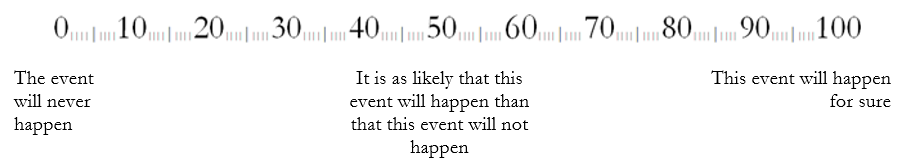


FILTER: The questions below are asked only to participants who are still active

- What is the probability that you will use a condom for your next paid sexual act? Value between 0 and 100

- What is the probability that you will use a condom for each of your next 10 paid sexual acts? Value between 0 and 100

- During your LAST 5 SEX ACTS, how many of them were protected sexual acts? Number of protected sex acts

***Taking of condoms***

Show the bowl containing 20 condoms to the respondent and READ: « The end of the interview is approaching. In this bowl there are condoms; do you want to take some? Please take only those you will use.”

- DO NOT READ: Please indicate if any condoms remain in the bowl. Yes/ No

- If she did not take all the condoms ask her why? Does not use condoms/ Does not have a bag to carry them/ Has no place where to hide them at home/ Does not like this quality of condom/ Does not need more condoms/ Other

- If other, specify: Text

- Please indicate how many condoms are left in the bowl.

***HIV test at the health center***

INTERVIEWER: You will read the following to the respondent: “I will now give you some information on the benefits of conducting HIV testing regularly. Taking a screening test is important in order to be quickly taken care of in case of HIV infection. HIV positive people can be healthy for many years before they develop symptoms and get sick, yet during this time, this person can transmit the virus to people with whom it has unprotected sex. If a person gets screened and is infected, she can take antiretroviral therapy that prevents the disease from developing, which improves her quality of life and prevents the transmission of the virus to other people.”

READ: “As you are currently in an STI center that offers voluntary and free testing, would you like to take advantage of your presence at the facility to know your HIV status?”

- Would you like to have a HIV test now at the facility? Yes/ No

If yes, ENUMERATOR : Refer the respondent to the midwife who will perform the pre-test counselling and testing.

 If no, ENUMERATOR: Thank the respondent for their participation and complete the interview. READ: "The interview is now over, we thank you for your time and we will give you the financial compensation that corresponds to the gains of the different tasks.”

**Additional outcomes** (using health facility logs)

- Did the respondent actually complete the screening test? Yes/ No

- If yes, what is the result of the test? Positive/ Negative

**Appendix S2.2: Setting of the experiment**


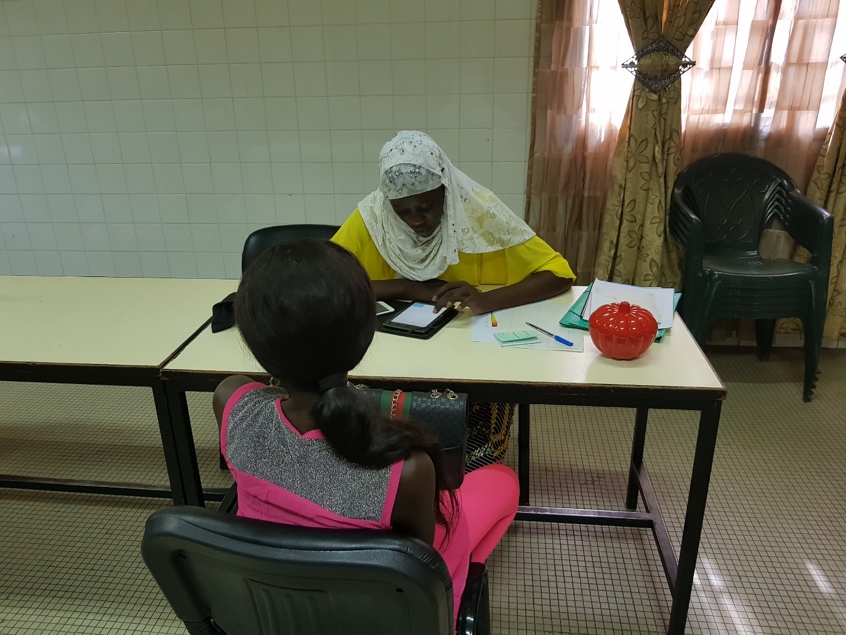


*Note:* The condoms were contained in the red bowl displayed on the table.

The participant did not know the content of the bowl until the end of the interview,

when she was offered the possibility to take some condoms.

**
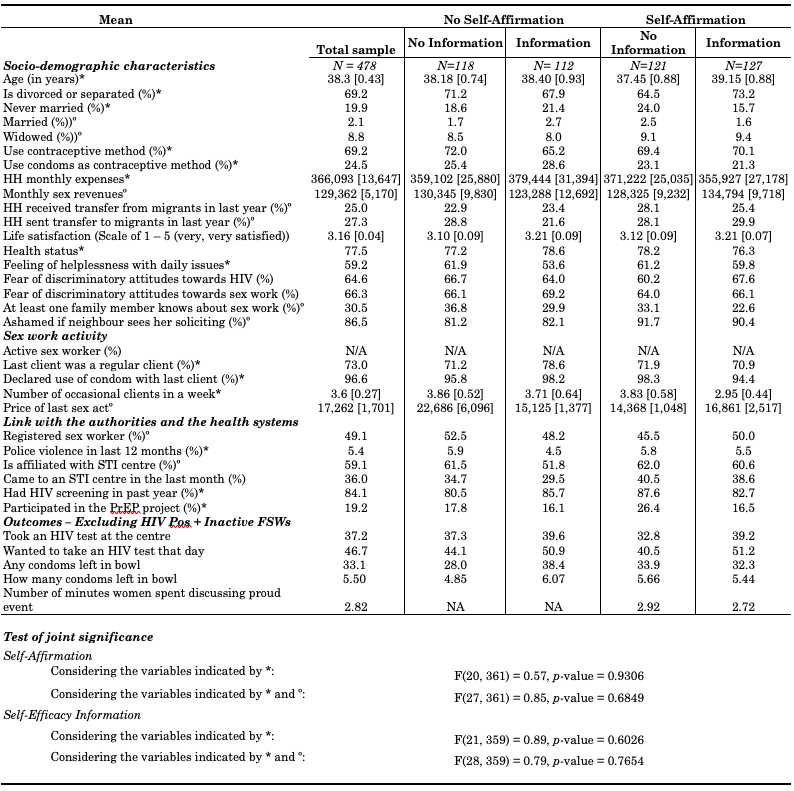
Appendix S3: Descriptive Statistics for Population Excluding HIV Positive and Inactive Female Sex Workers**

**Appendix S4: Average Marginal Effects for Specific Proud Events**

**Appendix S4.1 Excluding Inactive and HIV+ FSWs (Proud of Something Compared to Those Who Were Not Affirmed)**

*
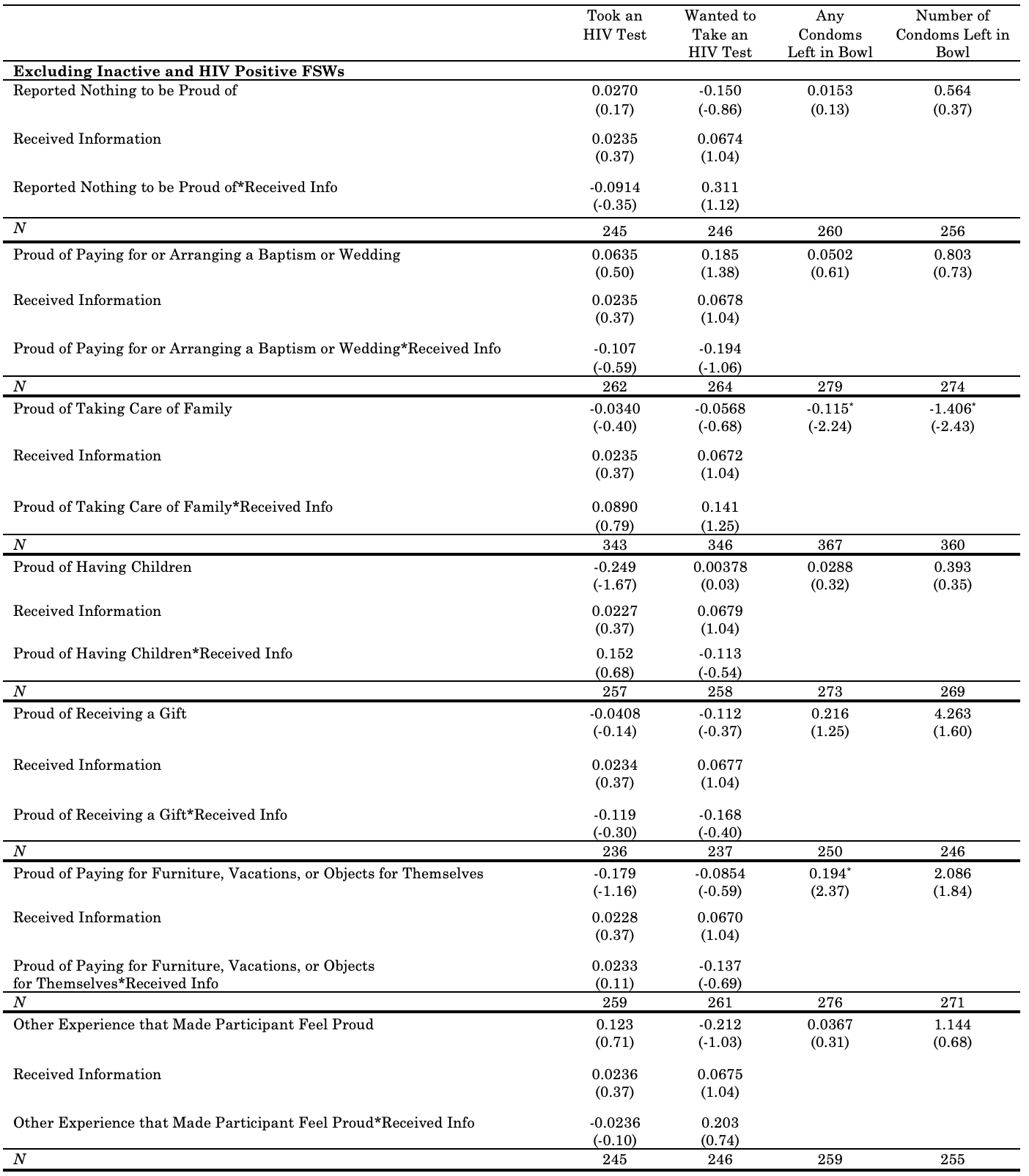
*

*Note.* Results exclude inactive sex workers, HIV+ FSWs. Columns (1), (2), and (3) are results from a logit regression and (4) are results from a tobit regression. Proud of something compared to people who were not affirmed. *t* statistics in parentheses. *** p<0.01, ** p<0.05, * p<0.1

**Appendix S4.2 Total Population (Proud of Something Compared to Those Who Were Not Affirmed)**

**
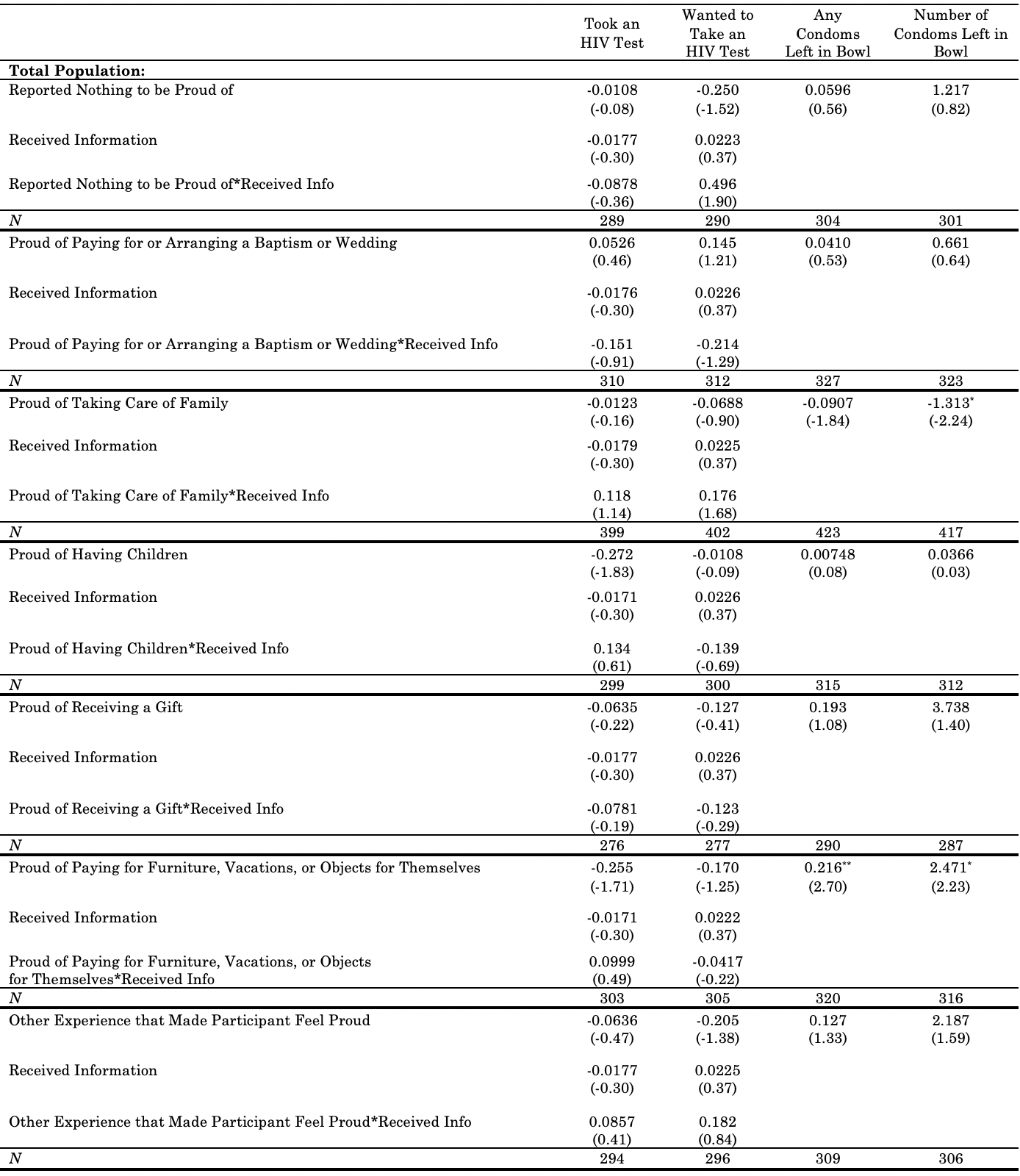
**

*Note.* Columns (1), (2), and (3) are results from a logit regression and (4) are results from a tobit regression. Proud of something compared to people who were not affirmed. *t* statistics in parentheses. *** p<0.01, ** p<0.05, * p<0.1

**Appendix S4.3 Excluding Inactive and HIV Positive FSWs (proud of something compared to those who are proud of something else)**

**
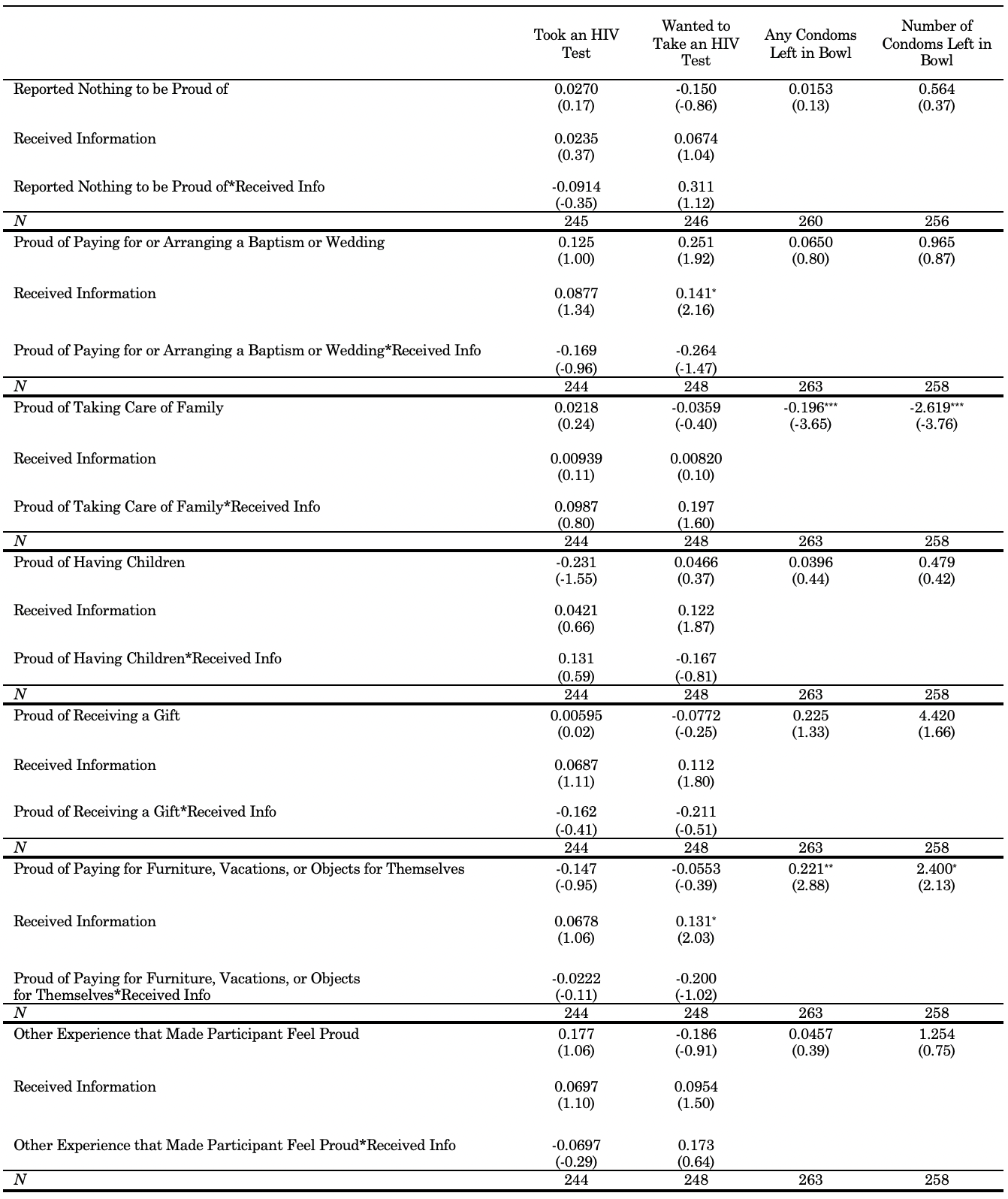
**

*Note.* Columns (1), (2), and (3) are results from a logit regression and (4) are results from a tobit regression. Proud of something compared to people who were proud of something else. *t* statistics in parentheses. *** p<0.01, ** p<0.05, * p<0.1

**Appendix S4.4 Total Population (proud of something compared to those who are proud of something else)**

**
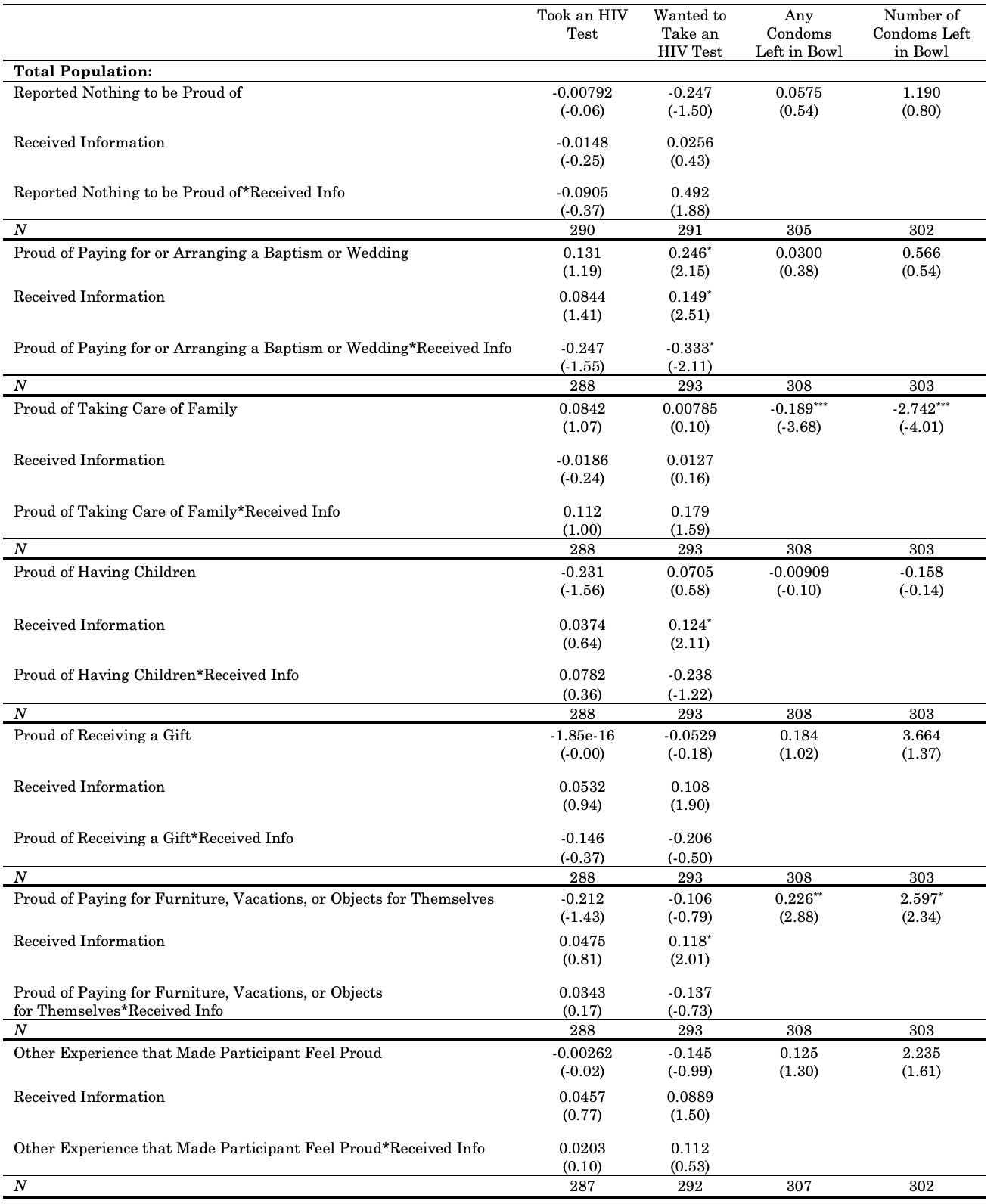
**

*Note.* Columns (1), (2), and (3) are results from a logit regression and (4) are results from a tobit regression. Proud of something compared to people who were proud of something else. *t* statistics in parentheses. *** p<0.01, ** p<0.05, * p<0.1

**Appendix S5: Information & No Information # Self-Affirmation*Discussion Length Predicted Probabilities (Average Marginal Effects)**

**Appendix S5.1 Excluding Inactive and HIV Positive FSWs**

|  | (1) | (2) | (3) | (4) |
| --- | --- | --- | --- | --- |
|  | Took an HIV Test | Wants to Take an HIV Test | Any Condoms Left in Bowl | Number of Condoms Left in Bowl |
| Received Information | 0.0816 | 0.150 |  |  |
|  | (0.49) | (0.89) |  |  |
|  |  |  |  |  |
| *Reference: Self-Affirmed & Discussed for 1 Min* | |  |  |  |
| Self-Affirmed & Discussed for 2 Min | 0.111 | 0.142 | -0.100 | -2.178 |
|  | (1.11) | (1.38) | (-0.91) | (-1.02) |
|  |  |  |  |  |
| Self-Affirmed & Discussed for 3 Min | 0.0380 | 0.136 | -0.0574 | -2.649 |
|  | (0.35) | (1.20) | (-0.47) | (-1.17) |
|  |  |  |  |  |
| Self-Affirmed & Discussed for 4 Min | 0.100 | 0.0355 | -0.169 | -4.665 |
|  | (0.73) | (0.26) | (-1.22) | (-1.92) |
|  |  |  |  |  |
| Self-Affirmed & Discussed for 5+ Min | 0.155 | 0.316^*^ | -0.249 | -5.161 |
|  | (0.94) | (1.99) | (-1.73) | (-1.82) |
|  |  |  |  |  |
| Received Information * Self-Affirmed | -0.00623 | -0.0114 |  |  |
|  | (-0.11) | (-0.20) |  |  |
| *N* | 244 | 248 | 248 | 248 |

*Note.* Results reported are the average marginal effects. The results exclude inactive sex workers and those who had previously tested positive for HIV. Columns (1), (2), and (3) are results from a logit regression and (4) are results from a tobit regression. *t* statistics in parentheses. *** p<0.01, ** p<0.05, * p<0.1

**Appendix S5.2 Total Population**

|  | (1) | (2) | (3) | (4) |
| --- | --- | --- | --- | --- |
|  | Took an HIV Test | Wants to Take an HIV Test | Any Condoms Left in Bowl | Number of Condoms Left in Bowl |
| Received Information | 0.109 | 0.120 |  |  |
|  | (0.66) | (0.73) |  |  |
|  |  |  |  |  |
| *Reference: Self-Affirmed & Discussed for 1 Min* | | | | |
| Self-Affirmed & Discussed for 2 Min | 0.105 | 0.0819 | -0.0752 | -1.831 |
|  | (1.07) | (0.77) | (-0.67) | (-0.83) |
|  |  |  |  |  |
| Self-Affirmed & Discussed for 3 Min | 0.0862 | 0.102 | -0.0805 | -3.088 |
|  | (0.80) | (0.87) | (-0.66) | (-1.33) |
|  |  |  |  |  |
| Self-Affirmed & Discussed for 4 Min | 0.134 | -0.0164 | -0.157 | -4.773 |
|  | (1.02) | (-0.12) | (-1.15) | (-1.92) |
|  |  |  |  |  |
| Self-Affirmed & Discussed for 5+ Min | 0.139 | 0.201 | -0.188 | -3.732 |
|  | (0.91) | (1.27) | (-1.23) | (-1.20) |
|  |  |  |  |  |
| Received Information * Self-Affirmed | -0.0164 | -0.0116 |  |  |
|  | (-0.30) | (-0.21) |  |  |
| *N* | 268 | 273 | 273 | 273 |

*Note.* Results reported are the average marginal effects. The results exclude inactive sex workers and those who had previously tested positive for HIV. Columns (1), (2), and (3) are results from a logit regression and (4) are results from a tobit regression. *t* statistics in parentheses. *** p<0.01, ** p<0.05, * p<0.1 **Appendix S6: Average Marginal Effects Excluding Inactive FSWs, HIV+ FSWs and FSWs Who Have Taken an HIV test in the Last 6 Months**

**
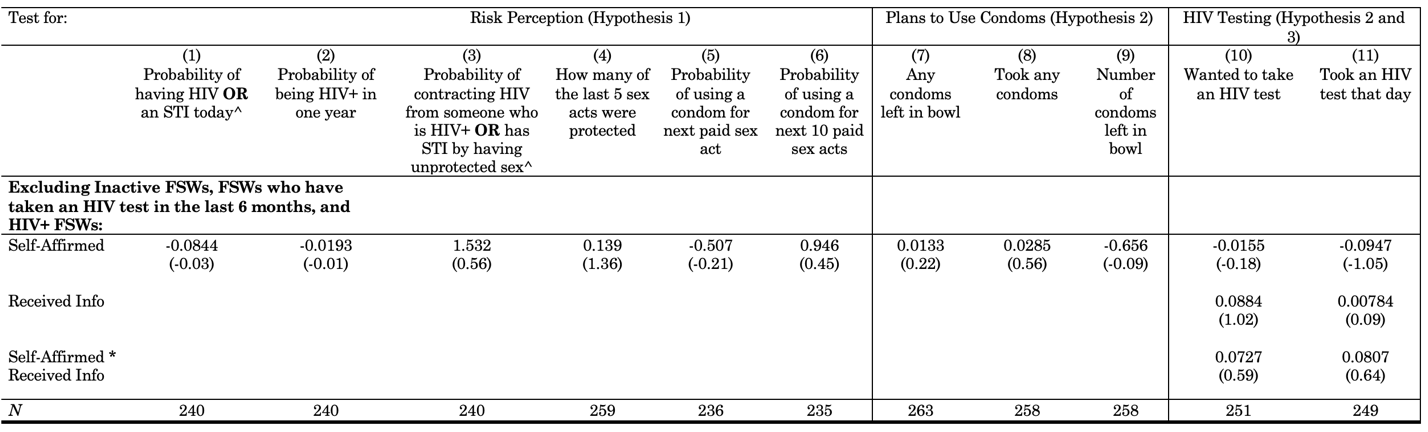
**

*Note.* ^ indicates two variables were averaged if they were highly correlated. Columns 1-6, and 9 are the average marginal effects from a tobit regression and columns 7-8 and 10-11 are the average marginal effects from a logit regression. *t* statistic in parentheses. *** p<0.01, ** p<0.05, * p<0.1

**Appendix S7: Average Marginal Effects (Registered and Unregistered FSWs)**


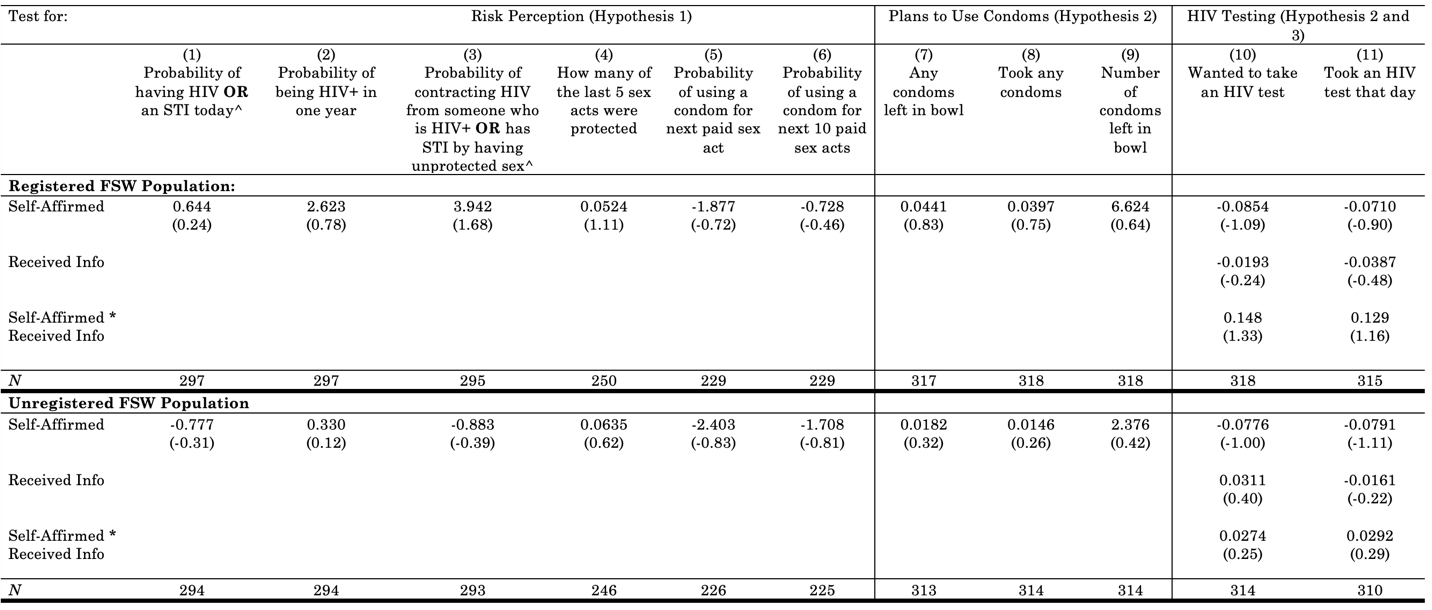


*Note.* ^ indicates two variables were averaged if they were highly correlated. Columns 1-6, and 9 are the average marginal effects from a tobit regression and columns 7-8 and 10-11 are the average marginal effects from a logit regression. *t* statistic in parentheses. *** p<0.01, ** p<0.05, * p<0.1
